# Curcumin induced oxidative stress attenuation by N-acetylcysteine co-treatment: a fibroblast and epithelial cell in-vitro study in idiopathic pulmonary fibrosis

**DOI:** 10.1186/s10020-019-0096-z

**Published:** 2019-06-13

**Authors:** L. R. Rodriguez, S. N. Bui, R. T. Beuschel, E. Ellis, E. M. Liberti, M. K. Chhina, B. Cannon, M. Lemma, S. D. Nathan, G. M. Grant

**Affiliations:** 1 Department of Biology, George Mason University, 10900 University Blvd, Manassas, VA 20110 USA; 2 Inova Advanced Lung Disease and Transplant Program, Inova Fairfax Hospital, 3300 Gallows Road, Falls Church, VA 22042 USA

**Keywords:** Curcumin, N-acetylcysteine, Pulmonary fibrosis

## Abstract

**Background:**

Idiopathic Pulmonary Fibrosis (IPF) is a fatal lung disease of unknown etiology with only two federally approved drug options. Given the complex molecular pathogenesis of IPF involving multiple cell types and multiple pathways, we explore the effects of a potential antifibrotic and antioxidant drug combination. Curcumin is a polyphenolic compound derived from turmeric with significant biological activity including a potential antifibrotic capacity. N-acetylcysteine (NAC) is a precursor to the antioxidant glutathione. To advance our understanding of these molecules, and to identify a clinical application, we present a small number of focused experiments that interrogates the effect of curcumin and NAC on pathways relevant to IPF in both fibroblasts and epithelial cells.

**Methods:**

Primary epithelial cell and fibroblasts isolated from patients with IPF were challenged with a combination treatment of NAC and curcumin. Evaluation of the antifibrotic potential and effect on oxidative stress was performed through QPCR gene expression analysis and functional assays including scratch tests, viability assays, and measurement of induced reactive oxygen species.

**Results:**

We demonstrate that curcumin alone does have antifibrotic potential, but that effect is accompanied by proapoptotic increases in oxidative stress. Coupled with this, we find that NAC alone can reduce oxidative stress, but that epithelial cell viability is decreased through this treatment. However, co-administration of these two molecules decreases oxidative stress and maintains high cell viability in both cell types. In addition, this co-treatment maintains an antifibrotic potential.

**Conclusions:**

These findings suggest a novel application for these molecules in IPF and encourage further exploration of this potential therapeutic approach.

## Introduction

Idiopathic pulmonary fibrosis (IPF) is the most common interstitial lung disease. Characterized by the overabundance of a highly active fibroblast population (Amara et al., 2010; Strieter & Mehrad, 2009), this fatal lung disease claims the lives of an estimated 41,000–82,000 people annually in Europe and the United States (Hutchinson et al., 2014). This is of particular concern for the world’s aging population as the incidence increases significantly with age (Raghu et al., 2006). The importance of this for developed countries cannot be overstated as the elderly population is expected to double over the next 25 years (Mora et al., 2017). Currently, a number of drugs are at various stages of development, but only two drugs have received FDA approval for the treatment of IPF (Mora et al., 2017).

The underlying cause of IPF is poorly understood; however, among the various theories of pathogenesis is a unifying observation of persistent cellular damage and stress accompanied by abnormal tissue repair (Datta et al., 2011). A key cellular player in both tissue repair and fibrosis, the fibroblast, has emerged as a potential target for small molecule intervention, including both FDA approved IPF drugs (Robalo-Cordeiro et al., 2017). The fibroblast is not the only cell that plays a significant role in disease. The hallmark failure in tissue repair is further exacerbated by an apoptotic cascade in the underlying epithelial cells which may serve to further propagate the wound response in a feedforward loop (Camelo et al., 2014). We suggest that while the fibroblast is a high value target for therapy, the use of small molecule intervention should consider the surrounding epithelium as a secondary adjunct target.

N-acetylcysteine (NAC), a precursor to the major antioxidant glutathione (Demedts et al., 2005), was a popular treatment option in the early 2000’s due to significant evidence demonstrating a decrease in oxidative stress burden in patients with lung fibrosis (Demedts et al., 2005; Behr et al., 1997; Meyer et al., 1994). However, in clinical trials, adding NAC to the standard of care in IPF resulted in mixed findings and failed to produce significant evidence that NAC alone improves lung function in mild to moderately impaired patients (Martinez et al., 2014). Most disturbingly, in 2012, a well-publicized clinical trial combining Prednisone, Azathioprine, and NAC in patients with IPF was prematurely stopped due to the adverse effects seen in the three-drug combination as compared to control (Network, 2012). Yet, even in this trial, the conclusion drawn for NAC was mixed; the patients taking NAC alone were allowed to complete the trial fully as they did not demonstrate the adverse effects seen in the combination arm (Martinez et al., 2014).

Curcumin is a hydrophobic polyphenol and the main active component of the spice turmeric. It has been used for thousands of years in Asian countries and traditional Ayurvedic medicine to inhibit scar tissue formation in open wounds (Gupta et al., 2012a; Gupta et al., 2012b). A significant application for curcumin in modern medicine has been highly elusive in spite of the large amount of effort and interest over the last half century (Gupta et al., 2012a). Over 65 US based clinical trials involving curcumin have been completed (Yuan & Du, 2018) with more than 50 in progress at the writing of this manuscript. While often characterized as having broad biological activities that can be applied to multiple diseases, the lack of significant translational success may be due to our poor understanding of the molecular mechanism.

Curcumin has been shown to have a broad range of antioxidant, antibacterial, antifungal, antiviral, anti-inflammatory, anti-proliferative, and pro-apoptotic properties (Aggarwal & Sung, 2009). Curcumin has also been reported to have anti-fibrotic capabilities in studies of wound healing, liver fibrosis and lung fibrosis models (Lin et al., 2009; Yao et al., 2012; Zhang et al., 2011; Smith et al., 2010; Chen & Zheng, 2008). At the molecular level, curcumin has been reported to play an anti-fibrotic role by modulating transcription factors such as transforming growth factor beta (Chen et al., 2013; Liu et al., 2016), platelet-derived growth factor, fibroblast growth factor and tumor necrosis factor alpha (Shishodia, 2013; Hua et al., 2013; Das & Vinayak, 2014), all of which are implicated in the pathogenesis of IPF. Due to these antifibrotic properties of curcumin, a number of investigators have hypothesized that this compound could serve as a possible therapeutic for IPF (Smith et al., 2010; Liu et al., 2016; Xu et al., 2007). These studies focus on the antifibrotic effects of curcumin and, like many other in-vitro studies, report a reduction in profibrotic responses when pulmonary fibroblasts are treated in isolation. However, these same studies report little significant improvement in bleomycin mouse models after curcumin treatment.

We have previously observed that treatment of fibroblasts in vitro with curcumin induces both an increase in reactive oxygen species (ROS) production (Rodriguez et al., 2018a), and apoptosis in primary fibroblasts but not in A549 alveolar epithelial cells (Bui, 2018). We hypothesize that increased oxidative stress may be a contributor to the pro-apoptotic properties of curcumin, and that alveolar epithelial cells may manage this burden more effectively possibly indicating that in the lung, curcumin is a fibroblast-specific drug. We further suggest that if curcumin-induced apoptosis is through ROS production, then co-treatment with a potent ROS scavenger may inhibit apoptosis. To explore this hypothesis, we investigated the effects of curcumin and NAC on IPF derived pulmonary fibroblasts and epithelial cells in vitro.

## Materials and methods

### Donor consent and internal review board approval

IPF lung tissue was obtained through Inova Fairfax Hospital (VA). All normal control lungs were obtained through the Washington Regional Transplant Community (WRTC). Appropriate written informed consent was obtained for each patient and donor by Inova Fairfax hospital and the WRTC. This study was approved by the Inova Fairfax Hospital Internal Review Board (IRB #06.083) and the George Mason University Human Subject Review Board (Exemption #5022). All experiments were performed in accordance with relevant guidelines and regulations.

### Specimen procurement/dissection and cell culture

The primary fibroblasts used in this study were isolated from human lungs procured in the operating room within minutes of explantation. The lungs were oriented from apex to base, and all samples used in this study were taken from the peripheral lower lobe of the lung. Fibroblasts were isolated from the lung tissue of four patients with advanced IPF (IPF-F) and four normal lungs (NHLF) using differential binding. Differential binding applied in this study is a modified protocol from that previously described (Emblom-Callahan et al., 2010; Rodriguez et al., 2018b). Samples were dissected into 1–2 mm^2^ pieces and subjected to enzymatic digestion in 0.4% collagenase P (Roche, Indianapolis, IN) complete media Dulbecco Minimal Essential Media (DMEM) containing 10% batch controlled fetal bovine serum (FBS), penicillin (100 I.U/ml), streptomycin (100 MCH/ml), amphotericin B (0.25 M.C.G./ml P/S/A) and 0.1% DNase1, at 37 °C and 5% CO_2_ for 2 h. The resulting material was passed through sterile cell filters (40, 100 μ nylon mesh) to remove undigested tissue and remaining cells were pelleted by centrifugation at 1000 g for 5 min. The pelleted cells were then suspended in complete media and seeded onto non-tissue culture plastic for 10 min at 37 °C and 5% CO_2_ to remove macrophages. The supernatant containing all unattached cells were then transferred to tissue culture treated plastic at 37 °C and 5% CO_2_ for 45 min. The attached fibroblast population was then vigorously washed with PBS to remove any unattached cells. This process was repeated twice more, and the final supernatant was transferred into a clean 15 ml conical for cell pelleting by centrifugation. This cell pellet was then resuspended in serum-free airway epithelial cell media (PromoCell) containing 1% antibiotic. The resuspended pellet containing primary epithelial cells was transferred to tissue culture plastic and placed into the incubator for continued culture. Media was replaced consistently every three days, and cells were passaged when confluency reached 70–80%. Analysis was carried out on cells within the 2–5 passage range.

### Quantitative real-time PCR (QPCR) analysis

To assay gene expression QPCR was carried out using cDNA generated from 1 μg of total RNA using iScript cDNA synthesis kit (Bio-Rad, Hercules, CA). QPCR was carried out using Quantifast SYBR Green PCR Kit (Qiagen). QPCR was carried out in triplicate using mRNA specific primers (Table 1) and normalized to 18S expression levels using the delta-delta CT method (Pfaffl, 2001).

**Table 1:** Primer Sequences

Gene	Forward Sequence	Reverse Seqeunce
18S	GATGGGCGGGGAAAATAG	GCGTGGATTCTGCATAATGGT
HIF1A	CCAACCTCAGTGTGGGTATAAG	CTGTGGTGACTTGTCCTTTAGT
SOD2	CCCAGAACAGGACAGAGAATG	AGGTGTAAGCCCACGTTTATC
CAT	GGTAACCCAGTAGGAGACAAAC	CGAGATCCCAGTTACCATCTTC
NRF2	TGCTCAATGTCCTGTTGC	GAGAGCCCAGTCTTCATTG
PCNA	TCCCACGTCTCTTTGGTGC	TCTTCGGCCCTTAGTGTAA
ACTA2	AGTTACGAGTTGCCTGAT	GAGGTCCTTCCTGATGTCAA
p53	AAGATGCAGGAACCGTCAGG	CCTGCGTCTGGAACTGGAAT
p21	GAGACTAAGGCAGAAGATGTAGAG	GCAGACCAGCATGACAGAT
CCND1	GTGCTGCGAAGTGGAAACC	ATCCAGGTGGCGACGATCT
COL1A1	GTCGAGGGCCAAGACGAAG	CAGATCACGTCATCGCACAAC

### Migration assessment through scratch test

To assay the in-vitro cell migration capability of each fibroblast cell line we used a modified protocol based on methodologies published by Liang et al. *(Liang et al.,* 2007*)*. Each cell line was grown to 70% confluency in 75 cm^2^ Falcon® tissue culture flasks prior to the start of the assay. Cells were then seeded in a Costar® 6-well tissue culture treated plate at a concentration of 300,000 cells per well. Each cell line was seeded in triplicate and, once the cells reached a confluency of 90%, the cell monolayer was scratched in a straight line with a p200 pipet tip (Fisher). Excess debris was removed by gentle washing of cells with 1 ml of growth medium followed by replacement of media with 1.5 ml of either fresh or treated growth media. Images of the scratch were captured at 4X magnification using an EVOS FL Auto Light Microscope (Life Technologies). Images were captured once every hour for 24 h. The scratch test images were analyzed using TScratch Version 1.0 (Gebäck et al., 2009).

### Apoptosis assay

Prior to each experiment, all cells were pre-treated in the same manner. Cells were grown to 90% confluency and serum-starved overnight. Cells were then seeded in triplicate at 5000 cells per well in a 96 well plate in complete media and allowed to attach overnight. Cells were then challenged for 24-h in varying concentrations of curcumin, NAC, or co-treatment as reported in the results section. Quantification of cell number was performed using the CellTiter 96® AQ_ueous_ Cell Proliferation Assay (Promega).

### Reactive oxygen species (ROS) determination

As previously mentioned cells were serum starved overnight prior to seeding in triplicate in 96-well plates at 10,000 cells/well. After overnight attachment, cells were incubated with 2′7’-dichlorofluorescin diacetate (DCFDA, Abcam) for 45 min in the dark. Cells were then challenge with curcumin, NAC or co-treatment for 2 h before fluorescent reading at Ex/Em 485/535.

### Statistical analysis

Statistical analysis was performed with Microsoft Excel using student t-tests. A corresponding *P*-values of less than 0.05 was considered statistically significant.

## Results

### Antifibrotic capacity of curcumin is attenuated in the presence of NAC

**Fig. 1:**
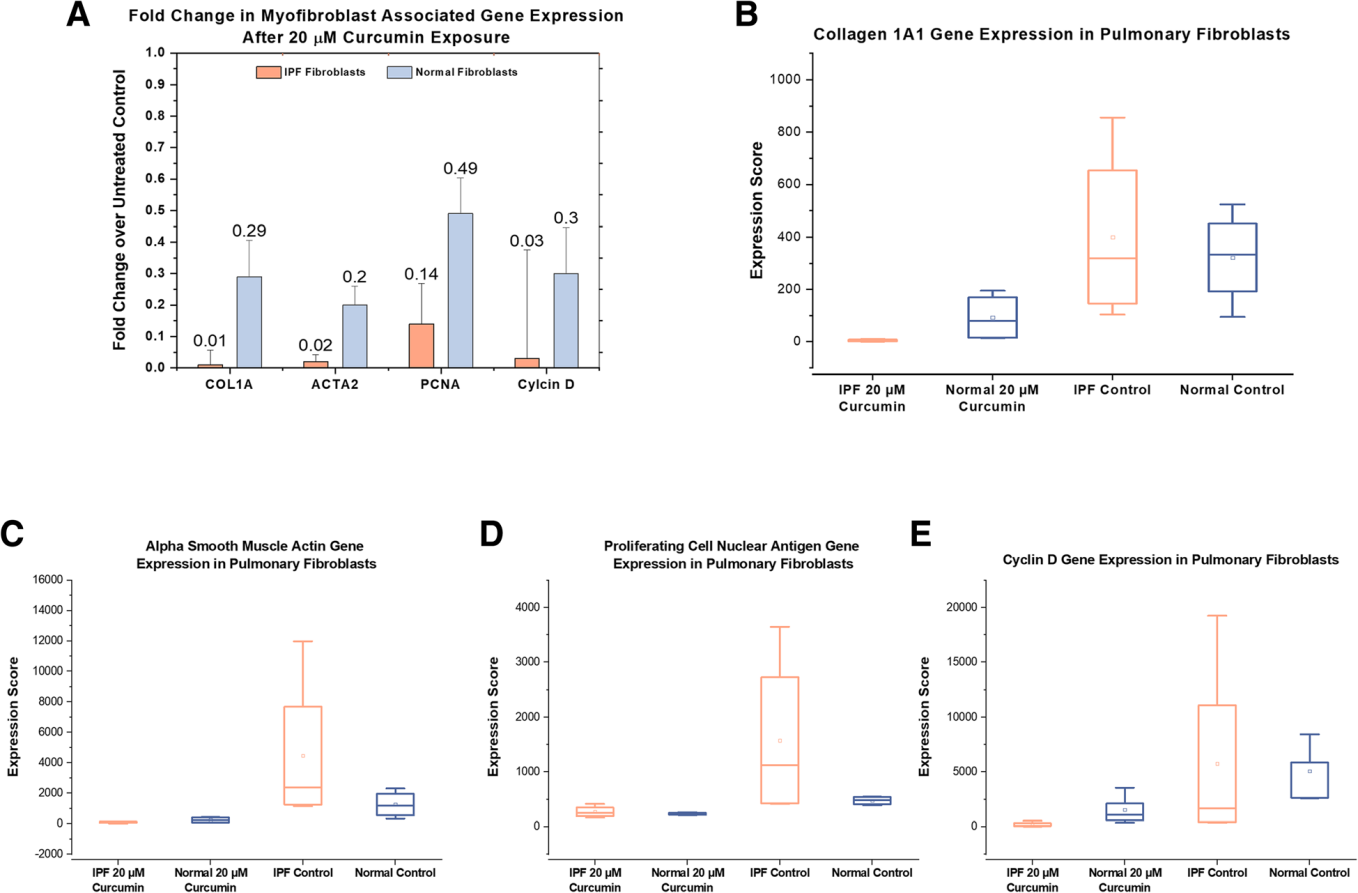
Curcumin treatment reduces myofibroblast associated gene expression in fibroblasts: (**a**) 24-h 20 μM curcumin treatment significant (*p* < 0.05) reduces gene expression of Collagen 1a1, Smooth Muscle Actin, Proliferating Cell Nuclear Antigen, and Cyclin D in both IPF-F (*n* = 4) and NHLF (*n* = 3) (**b**-**e**) Heterogeneity of gene expression before curcumin treatment is observed for all four genes in IPF-F but not in NHLF. Additionally this heterogeneity is absent after 20 μM curcumin treatment in both cell types. * indicates *p* value < 0.05 ** indicates *p* value < 0.005

To confirm the observation that curcumin can inhibit the myofibroblast phenotype in pulmonary fibroblasts (Smith et al., 2010) we exposed primary NHLF and IPF-F to 20 μM curcumin for 24 h (Fig. 1). We observe significant decreases in the expression of myofibroblast activation and proliferation associated genes in both the NHLF and IPF-F (Fig. 1a). Initial analysis of the data seemed to indicate that the antifibrotic effect of curcumin on IPF-F was more pronounced as the gene expression reduction was greater in IPF-F. However, this was not statistically significant and is likely attributable to the significant heterogeneity in the gene expression profile observed in IPF-F as compared to NHLF (Fig. 1b-e).

**Fig. 2:**
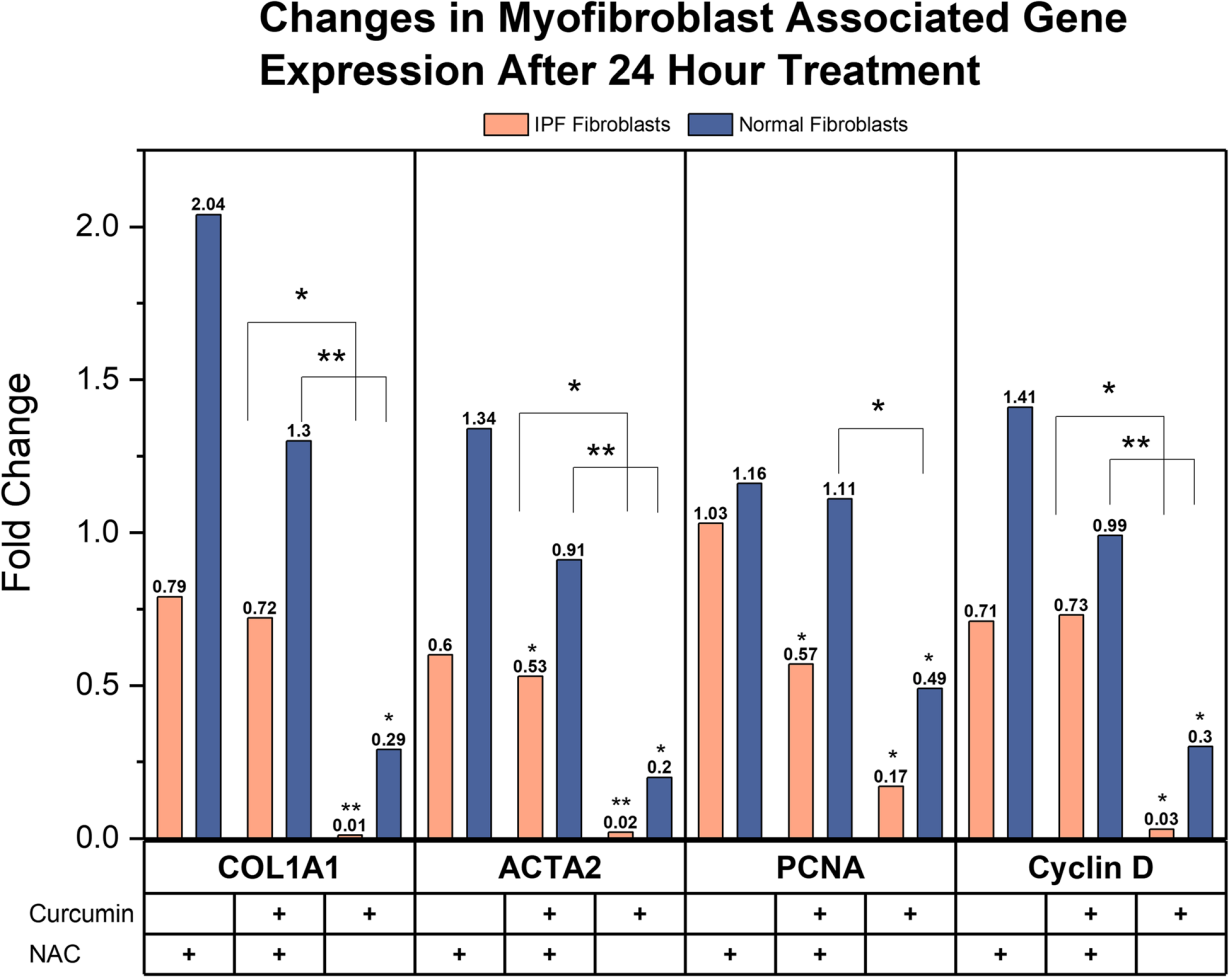
Effects of 24-h 10 mM NAC challenge and 24-h combination 40 μM curcumin/10 mM NAC challenge on myofibroblast associated gene expression in fibroblasts: 10 mM NAC alone or co-treatment with 40 μM curcumin and 10 mM NAC does not induce significant changes to gene expression of Collagen 1a1 (COL1A1), Smooth Muscle Actin (ACTA2), Proliferating Cell Nuclear Antigen (PCNA), and Cyclin D in NHLF (n = 3) as compared to untreated controls. Co-treatment in IPF-F (n = 4) induces significant decreases in Smooth Muscle Actin and Proliferating Cell Nuclear Antigen, but there is no significant change in Collagen 1A1 and Cyclin D as compared to untreated controls. When comparing treatment conditions there is a significant increase in the capacity of 20 μM curcumin alone to reduce gene expression of COLA1A, ACTA2 and Cyclin D in both IPF-F and NHLF as compared to co-treatment with 10 mM NAC. This significant trend is also observed for PCNA in NHLF. * indicates *p* value < 0.05 ** indicates *p* value < 0.005

We next explored the effect of NAC on the antifibrotic effect of curcumin (Fig. 2). The co-administration of curcumin and NAC continued to demonstrate a reduction in Smooth Muscle Actin (ACTA2) and Proliferating Cell Nuclear Antigen (PCNA) in IPF-F as compared to untreated control, however the expression of Collagen 1A1 (COL1A1) and Cyclin D (CCND1) was not significantly altered. This effect was not observed in the NHLF. While NAC alone did not have a significant effect on the expression of this gene profile in either fibroblast population, we do note that the antifibrotic effect of curcumin as measured by our panel was significantly attenuated by the addition of 10 mM NAC (Fig. 2). We also report, that in our co-therapy, there was an increased concentration of curcumin from 20 μM to 40 μM for induction of antifibrotic effects.

### IPF fibroblast migration inhibition by curcumin is attenuated in the presence of NAC

**Fig. 3:**
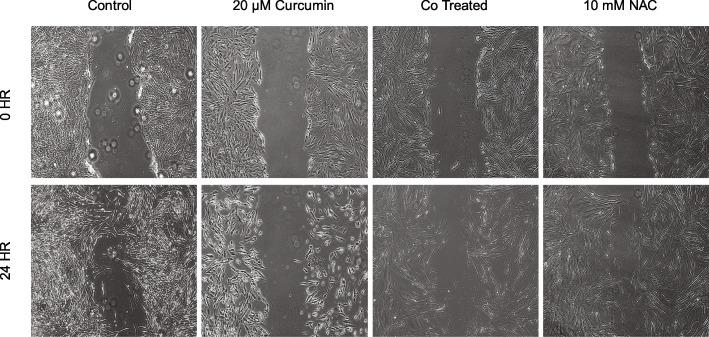
Representative scratch test results in IPF-F after 24-h treatment with NAC and curcumin: 20 μM curcumin reduces wound closure in IPF-F as compared to untreated control. 10 mM NAC does not have a significant effect on wound closure rate. Combination treatment with 40 μM curcumin and 10 mM NAC results in decreased wound closure as compared to untreated control but is attenuated in comparison to 20 μM curcumin alone. Morphological changes observed in the curcumin treatment are also attenuated in the combination treatment

**Fig. 4:**
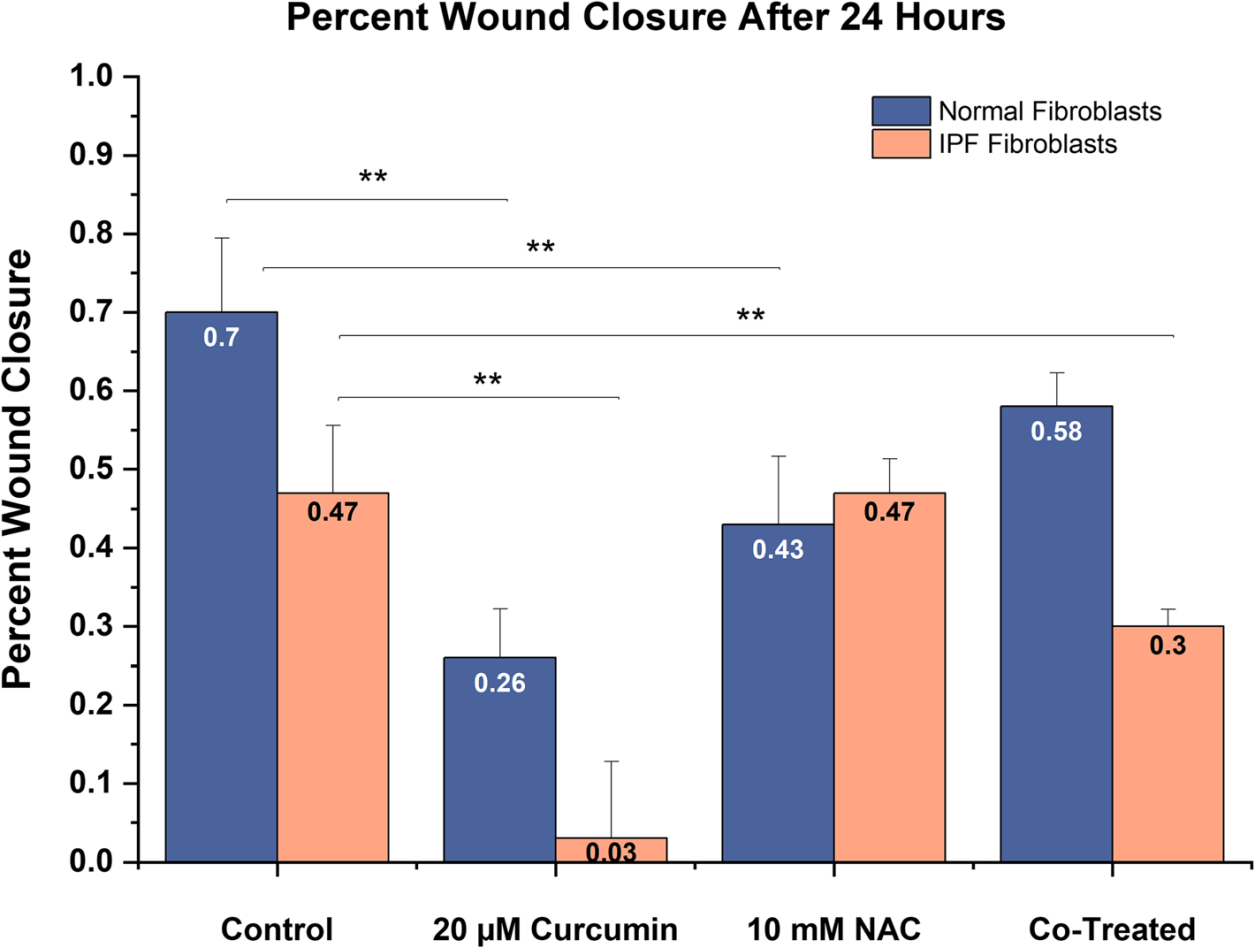
Quantification of wound closure data in IPF (n = 4) and NHLF (*n* = 3) after challenge: Significant reduction in 24-h wound closure rate is observed in IPF-F after 20 μM curcumin treatment and co-treatment with NAC. This same result is observed after 20 μM curcumin in NHLF and 10 mM NAC challenge. Co-treatment with NAC and 40 μM curcumin does not induce a significant reduction in wound closure rate as compared to untreated control. ** indicates *p* value < 0.005

To assess the functional effect of curcumin on the migratory capability of the fibroblast we performed a 24-h scratch test on IPF-F and NHLF (Figs. 3 and 4). We report a significant decrease in the wound closure rate of both IPF-F and NHLF in the presence of curcumin alone. Interestingly we also note that NAC alone reduced the migratory capability of NHLF (Fig. 4). However, the co-treatment of normal and IPF-F with NAC and curcumin does not inhibit the migratory capacity of the fibroblasts to the same degree as curcumin alone. In fact, we observe no significant change in wound closure for NHLF in the presence of the co-therapy as compared to control. There remains, however, an attenuated migratory inhibition on IPF-F in our co-therapy.

### Curcumin induced apoptosis of pulmonary epithelial and fibroblast cells is inhibited in the presence of NAC

**Fig. 5:**
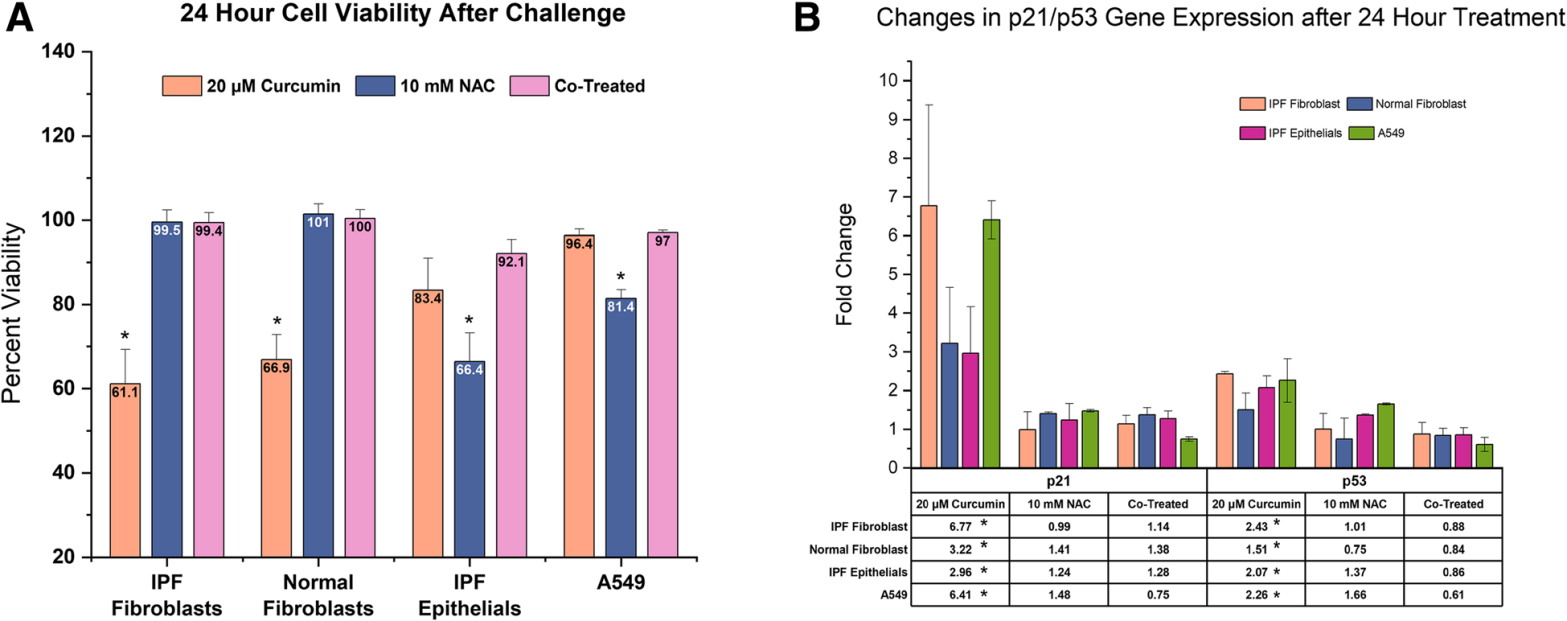
Epithelial and fibroblast cell viability and DNA damage response gene expression after 24-h challenge: (**a**) 20 μM curcumin treatment induces reduced viability in IPF-F (*n* = 4) and NHLF (*n* = 3) with significant effect on IPF epithelial cells (IPF-E *n* = 3) or A549 epithelial cells (n = 3). 10 mM NAC treatment induces reduced viability in epithelial cells but no significant effect in fibroblasts. Combination 10 mM NAC and 40 μM curcumin does not induce any change in cell viability as compared to untreated controls. (**b**) 20 μM curcumin induces significant increases in gene expression of p53 and p21 in all cell types. 10 mM NAC alone and in combination with 40 μM curcumin does not result in significant change to this gene expression in either epithelial or fibroblast cell lines. * indicates *p* value < 0.05

We, and others, have previously observed dose dependent increase in fibroblast apoptosis in the presence of curcumin (Zhang et al., 2011; Bui, 2018). We test a sublethal, high dose curcumin treatment to evaluate effect on epithelial cell viability (Fig. 5a). After 24-h curcumin exposure, we observed a significant reduction in NHLF and IPF-F viability but did not see a significant reduction in either A549 or primary epithelial cell viability. Treatment with 10 mM NAC alone had no effect on fibroblast viability, however this concentration did present reduced viability in epithelial populations. Co-treatment of all cell populations with curcumin and NAC had no significant effect on cell viability. Coupled with these data we report a concurrent increase in p21 and p53 gene expression after 20 μM curcumin treatment (Fig. 5b). Finally, we observed, no significant changes of p21 or p53 expression in any cell type in the NAC or co-treatment group.

### Curcumin induced oxidative stress is alleviated by NAC co-treatment

**Fig. 6:**
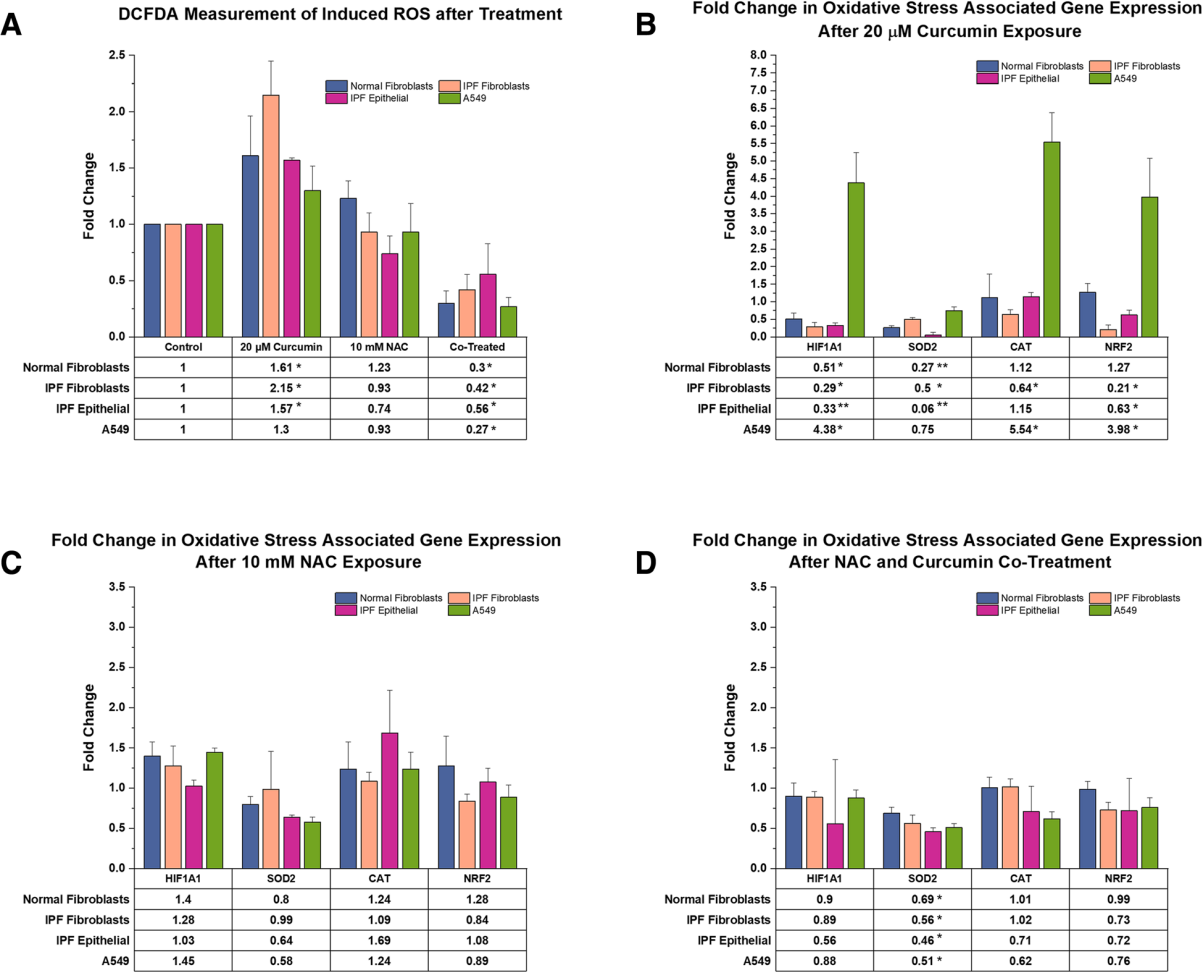
Induction of ROS and change in Oxidative Stress Associated gene expression after 24-h challenge in both epithelial and fibroblast cell lines: (**a**) 20 μM curcumin induces significant increases in ROS generation in all primary cell lines (IPF-F n = 3, NHLF n = 3, IPF-E n = 3), but no significant increase in A549 cells (n = 3). 10 mM NAC treatment has no significant induction of ROS, however co-treatment with 10 mM NAC and 40 μM curcumin results in a significant decrease in ROS in all cell types. (**b**) Challenge with 20 μM curcumin results in a decreased expression of Hypoxia inducible factor 1α (HIF1) and Superoxide Dismutase 2 (SOD2) in IPF-F (n = 4), NHLF (n = 3), IPF-E (*n* = 3). Expression of Catalase (CAT) is decreased in IPF derived fibroblasts while Nuclear Factor erythroid 2-related factor 2 (NRF2) expression is decreased in IPF-F and epithelial cells. A549 cells demonstrate an increase in expression of HIF1, CAT, and NRF2 after challenge with 20 μM curcumin. (**c**) 10 mM NAC challenge results in a significant decrease in SOD2 expression in primary IPF epithelial cells and A549s but no effect on any other gene in the assayed cells. (D) Co-treatment with 10 mM NAC and 40 μM curcumin induces decreased SOD2 expression in all cell types. * indicates *p* value < 0.05 and ** indicates *p* value < 0.005

Many of the reported health benefits attributed to curcumin come from the suggestion that it serves as a potent antioxidant (Hewlings & Kalman, 2017; Jha et al., 2015; Rahmani et al., 2018). To asses this we measured induced reactive oxygen species (ROS) in our cells after exposure to curcumin, NAC, and combination of the two (Fig. 6a). After 20 μM curcumin challenge we observe significant increases in ROS within all primary cells, and no significant increase was observed in the A549 populations. Treatment with 10 mM NAC alone did not induce any significant change in the measurable levels of ROS species. Interestingly, combined treatment with NAC and curcumin resulted in levels of ROS that are significantly reduced in all populations as compared to uninduced controls. In addition to the generation of ROS species, we also report the changes in an oxidative stress response gene panel consisting of Hypoxia Inducible Factor 1α (HIF1), Superoxide Dismutase 2 (SOD2), Catalase (CAT), and Nuclear factor-like 2 (NRF2). After curcumin challenge we observed significantly decreased expression of nearly all genes of this panel in both IPF-F and IPF epithelial cells. In NHLF we also see a significant reduction in expression of HIF1 and SOD2, but no concurrent change in NRF2 and catalase. A549 gene expression deviates from this pattern with significant increases in expression of HIF1, NRF2, and catalase. In contrast with these results, the challenge of these cells with NAC alone resulted in decreased expression of one gene, SOD2, in the epithelial cells only. Finally, combination treatment resulted in significant decreases in this same gene, SOD2, across all cell types with no change to any other gene within our panel.

## Discussion

In this study we sought to further explore the antifibrotic potential of curcumin and the effects that this molecule has on the IPF epithelial cell. We initially focused on the effects of curcumin on the myofibroblast phenotype to confirm previous studies and to establish a baseline from which to compare our later experiments. We observed that 24-hr treatment with curcumin was effective at reducing both the expression of myofibroblast associated genes such as COL1A1 and PCNA (Fig. 1), as well as reducing the migratory capability of the fibroblasts (Fig. 4). This observation confirmed previous reports (Lin et al., 2009; Xu et al., 2007), however we also noted that expression of p53 was significantly increased in these fibroblasts (Fig. 5b). These data, coupled with recent fibroblast studies reporting curcumin dose dependent increases in apoptotic markers (Zhang et al., 2011), lead us to hypothesize that oxidative stress may be playing a role in the antifibrotic properties of curcumin.

With oxidative stress as a major stressor in all cells in the IPF lung, we included both epithelial cells and fibroblast in our investigations. Measurement of ROS generation in these cells after curcumin challenge demonstrated a significant induction of ROS within the primary cells, however the immortal alveolar type II cell line A549 proved resistant (Fig. 6a). In addition to this, we noted a decrease in cell viability in the primary cells that was absent in A549 (Fig. 5a). These data indicate that increased oxidative stress induced by curcumin may be activating an apoptotic cascade in the primary cells. In support of this hypothesis, we note that gene expression of the DNA damage response protein p53 is increased in all cell lines. Concurrently, the transcriptional target of p53, and an S phase regulator - p21, is also increased in all cells. A549 cells do seem to contradict our hypothesis as the increase in this same gene expression is not accompanied by apoptosis. We suggest that the high basal expression of the p53 inhibiting protein MDM2 in A549 cells may be the source of this discrepancy (Liu et al., 2017). This indicates that manipulation of the MDM2/p53 pathway may be a viable mechanistic approach to reduce curcumin induced apoptosis in primary cell lines. Given our interest in IPF, we did not further explore this A549 pathway and rather, chose to alleviate the oxidative stress through NAC co-treatment.

Keeping in mind that a significant benefit of curcumin in IPF is its antifibrotic properties, we first explored the effect of a curcumin/NAC co-treatment on fibroblasts. Exposure to 20 μM curcumin resulted in a decreased myofibroblast phenotype (Fig. 1), but combined exposure with 10 mM NAC did not result in the same effect as measured by our gene expression analysis. Thus we increased the curcumin dosage to 40 μM, a level that has previously been demonstrated to be toxic (Bui, 2018). To our surprise, at 40uM curcumin dosage in combination with NAC did not induce apoptosis (Fig. 5a) and demonstrated antifibrotic capabilities (Figs. 2, 3 and 4) albeit at a reduced level as compared to curcumin alone. In addition to this finding we also observed that the co-treatment inhibited NAC induced apoptosis in epithelial cells (Fig. 5a).

Our hypothesis that reduction in curcumin induced oxidative stress would prevent apoptosis was supported by both the viability study and the reported reduction in ROS after NAC co-treatment (Fig. 6a). With these data in mind, we were interested in the genetic regulation of oxidative stress response genes in the presence of our two small molecules. When challenged with curcumin alone, all primary cell lines demonstrated a decreased expression of our oxidative stress panel (Fig. 6b). As with many other experiments, A549 proved an outlier with an increase in three of the four selected genes. Given our findings that curcumin induces ROS generation in these cells, these data indicate that the increased oxidative stress burden in primary cells is not accompanied by the robust response necessary to manage said burden. The addition of NAC co-treatment did not have a significant effect on the expression of most genes in the panel but given that this co-treatment did alleviate ROS generation we did not expect to see an increase in these genes. The one exception to this was the expression of SOD2. Our data indicates that SOD2 gene expression is tightly regulated by ROS generation. Given that this is the oxide dismutase found primarily in the mitochondria and, that mitochondria are a primary site for ROS generation, these data were complimentary.

A driving rationale for applying curcumin in IPF was the hypothesis that, in the lung, curcumin may be a fibroblast specific compound. Our study determined this to be somewhat untrue, but we suggest that our findings begin to develop a new paradigm for the application of curcumin and NAC in IPF. We report that, as is the case for many antioxidant molecules (Garry et al., 2009; Rietjens et al., 2002), curcumin has both a prooxidant and antioxidant capacity (Fig. 6). In primary epithelial cells and fibroblasts, curcumin inhibits the oxidative stress response, while in A549s curcumin induces a strong oxidative stress response. However, it is also clear that this stress response is, in part, the result of the capacity of A549s to deal with an increased ROS burden. We also confirm that curcumin has antifibrotic properties, though we do present evidence that the effect may be exaggerated by the induction of apoptosis through the p21/p53 cascade. Finally, these data reinforce that NAC is effective in reducing oxidative stress in pulmonary cells which is of significant therapeutic value where ROS production is induced.

We note that the concentration of both curcumin and NAC used in this study are at high in-vitro concentrations. Given the relatively low bioavailability of these compounds this is a significant concern in future translational studies. However, the clinical history of both compounds demonstrates that NAC and curcumin are well-tolerated compounds that can be given at high dosages without major secondary complications (Gupta et al., 2012b; Bando et al., 2010; Sanguinetti, 2015). We also acknowledge that a weakness to our study lies in our lack of data on varied concentrations in the co-treatment. Further studies will focus on varying concentrations of these compounds and investigating modifications or alternatives that may increase the bioavailability of this therapy such as the application of the NAC sister drug NACA (Aldini et al., 2018).

**Fig. 7:**
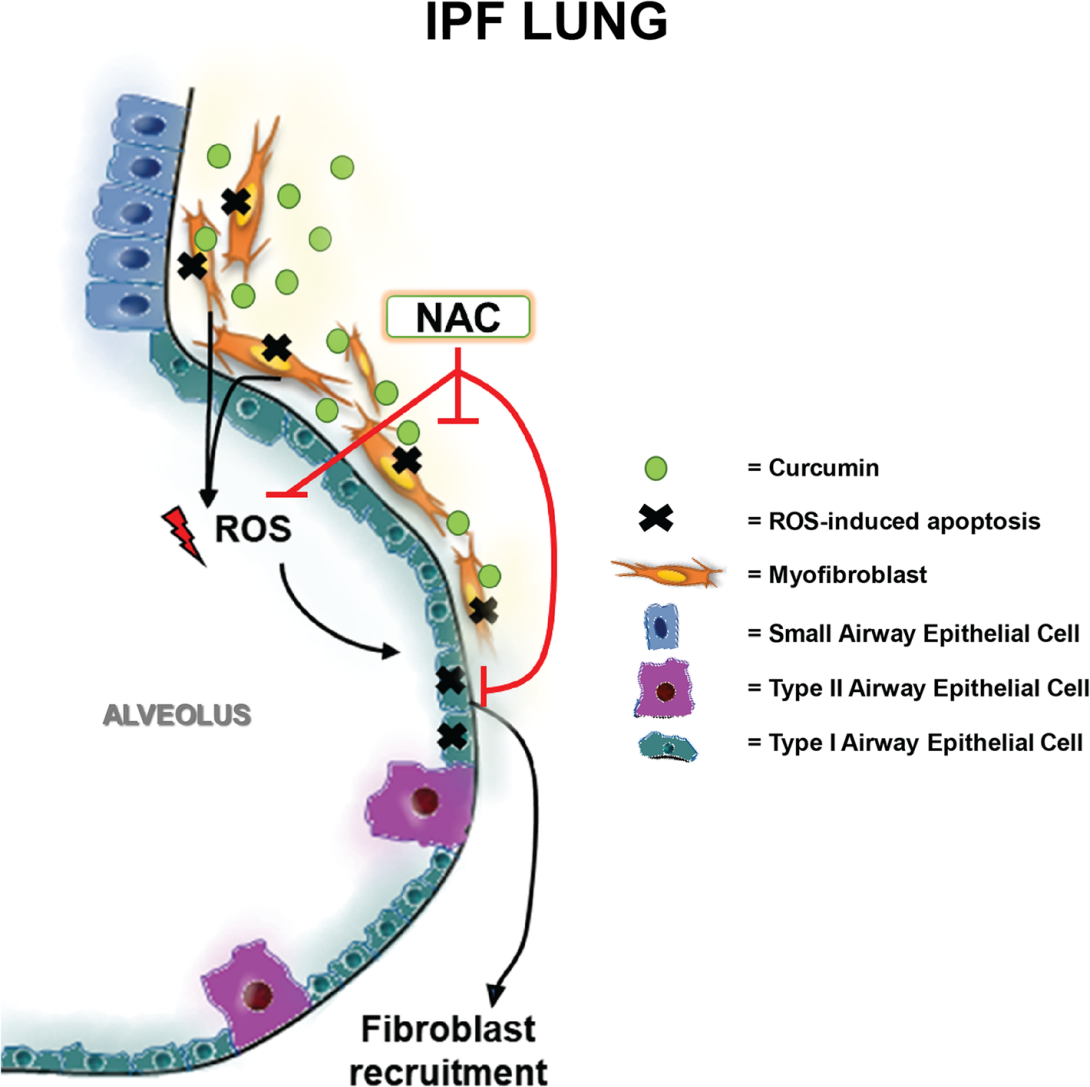
Hypothetical Molecular Model of Curcumin and NAC Co-Treatment in IPF: Curcumin induces ROS mediated apoptosis in myofibroblasts releases excess ROS into the microenvironment. As the IPF lung is an oxidative stress rich environment the excess ROS further damages epithelial cells in the lung. This propagates the wound healing response and may further induce fibrosis in a classical IPF feedforward loop. The introduction of NAC co-treatment attenuates fibroblast apoptosis and alleviates ROS induced oxidative stress in epithelial cells. In turn this prevents additional fibroblast recruitment. Deduction of optimal in-vivo co-treatment concentrations may result in significant antifibrotic potential for therapeutic application

These findings indicate that the application of curcumin alone is an ineffective treatment option for use in IPF. The apoptotic effect of induced ROS is of significant concern in IPF, particularly given the high levels of oxidative stress already found in patient lungs. Conversely, the alleviation of oxidative stress through NAC therapy alone is not a sufficient therapeutic approach. Our findings indicate that it may be possible to use these two treatments in combination, to elicit both the antifibrotic response and protect the surrounding epithelium from ROS induced apoptosis (Fig. 7). The co-treatment of our cells with NAC did mitigate the antifibrotic potential of curcumin and; it is possible that increased concentrations of curcumin may overcome this attenuation, however it is just as likely that this will result in overwhelming ROS generation. We suggest that further investigation into the oxidative stress generated by curcumin in pulmonary cells may aid in the elucidation of key pathways that can be manipulated to inhibit apoptosis and maintain high antifibrotic potential.

## Conclusions

In conclusion, the heterogeneity of IPF presents a significant challenge in the discovery of novel therapeutic approaches. Our findings suggest a novel combination of two molecules that have alone demonstrated a capacity to alleviate elements of the disease processes found in IPF. Perhaps the key to treating IPF is not to strongly inhibit a single disease process, but instead attempt to alleviate multiple aberrant pathways through drug combinations.

## Data Availability

All data generated or analyzed during this study are included in this published article.

## Abbreviations

ACTA2: Alpha Smooth Muscle Actin
CAT: Catalase:
CCND1: Cyclin D
COL1A1: Collagen 1A1
DMEM: Dulbecco Minimal Essential Media
HIF1: Hypoxia Inducible Factor 1α
IPF: Idiopathic Pulmonary Fibrosis
IPF-F: IPF Fibroblast
NAC: N-acetylcysteine
NHLF: Normal Human Lung Fibroblast
NRF2: Nuclear factor-like 2
PCNA: Proliferating Cell Nuclear Antigen
QPCR: Quantitative Polymerase Chain Reaction
ROS: Reactive Oxygen Species
SOD2: Superoxide Dismutase 2
